# Clinical Response of Metastatic Breast Cancer to Multi-targeted Therapeutic Approach: A Single Case Report

**DOI:** 10.3390/cancers3011454

**Published:** 2011-03-17

**Authors:** Christian Meiners

**Affiliations:** Gautinger Straße 3b, D-82234 Wessling, Germany; E-Mail: Meiners-Frankfurt@T-Online.de

**Keywords:** breast cancer, chemotherapy, respiratory chain, Warburg effect, inflammatory chain, partial remission

## Abstract

The present article describes the ongoing (partial) remission of a female patient (41 years old) from estrogen receptor (ER)-positive/progesterone receptor (PR)-negative metastatic breast cancer in response to a combination treatment directed towards the revitalization of the mitochondrial respiratory chain (oxidative phosphorylation), the suppression of NF-kappaB as a factor triggering the inflammatory response, and chemotherapy with capecitabine. The reduction of tumor mass was evidenced by a continuing decline of CA15-3 and CEA tumor marker serum levels and ^18^FDG-PET-CT plus magnetic resonance (MR) imaging. It is concluded that such combination treatment might be a useful option for treating already formed metastases and for providing protection against the formation of metastases in ER positive breast cancer. The findings need to be corroborated by clinical trials. Whether similar results can be expected for other malignant tumor phenotypes relying on glycolysis as the main energy source remains to be elucidated.

## 1. Introduction

Since Richard Nixon declared war on cancer about 30 years ago, much efforts have been made in order to overcome this dreadful disease. Enormous financial resources have been invested in cancer research in the last three decades, yet most metastasized solid malignant tumors are still considered incurable. Chemotherapy has been shown to be a potent (long lasting) treatment option against only a few solid cancers including testis cancer. The overall contribution of curative and adjuvant cytotoxic chemotherapy was assessed to be 2.3% in Australia and 2.1% in the United States of America with a five-year survival in adults based on data for 1998 [1]. Under chemotherapy, cancer cells can gradually develop drug resistance that is acquired, for instance, by overexpression of transporter proteins (e.g., those belonging to the ATP-binding cassette type) [2,3] and fractionation of the cancerous stem cells [4] (which are less sensitive to exposure to cytostatics than more differentiated cancer cells), plus AKT [5,6] and NF-kappaB [7,8] overexpression as a compensatory response to administered cytotoxic drugs. Likewise, induced hypoxia may act as protective shield against tumor eradication by chemotherapeutics and radiation due to alterations of gene expression profiles related to hypoxia, which result in the inhibition of apoptosis [9].

On the other hand, a plethora of “alternative” cancer therapies have been developed and applied in the past. Here, we report on a combination treatment, including chemotherapy, bisphosphonates, and complementary measures, aiming at the normalization of the cellular metabolism, vascular angiogenesis, cell life cycle, and cell proliferation activity.

## 2. Experimental

### 2.1. Chemicals/Dietary Supplements

Super Ubiquinol CoQ10, Life Extension, article nr. 01426, USA: www.lefeurope.com

Vitamin B2, tablets, 10 mg, Jenapharm^®^, Mibe GmbH, Germany

Vitamin B3, capsules, 54 mg, Allpharm, Germany, PZN 6605862

5-Loxin^®^capsules, 75 mg, (std. for acetyl-11-keto-β-boswellic acid (AKBA), minimum 30% on dry basis), Life Extension, article nr. 00939, USA, www.lefeurope.com

Linseed oil, Linosan Leinöl, Heirler Cenovis GmbH, D-78303 Radolfzell, Germany

Bio-Kefir, Andechser Natur, 1,5% fat, containing L(+) dextrorotatory lactic acid, Andechser Molkerei Scheitz GmbH, D-82346 Andechs, Germany, www.andechser-molkerei.de

Bio-Yoghurt, Andechser Natur, 0,1% fat, containing L. acidophilus and B. bifidus, Andechser Molkerei Scheitz GmbH, D-82346 Andechs, Germany, www.andechser-molkerei.de

Flaxseed, freshly ground

EPA/DHA: Mega EPA/DHA, capsules, Life Extension, article nr. 00625

Sodium selenite, Selenase^®^200 XXL, 200 μg selenium, biosyn Arzneimittel GmbH, D-70734 Fellbach, Germany

L-Carnitine: Multinorm^®^ L-Carnitin aktiv, 250 mg L-carnitin plus 3 μg Vitamin B12, Sankt Pirmin® Naturprodukte GmbH, D-55218 Ingelheim, Germany

L-Carnitine, 300 mg capsules: Altapharma, Germany

Zinc, Unizink^®^ 50, 50 mg zinc-bis(hydrogen-DL-aspartat), Kohler Pharma GmbH, D-64665 Alsbach-Hähnlein, Germany, PZN-3441621

Ibandronat Bondronat^®^, 6 mg/6 mL concentrate, Roche Pharma AG, D-79639 Grenzach-Wyhlen, Germany

Capecitabine, Xeloda^®^, Roche Pharma AG, D-79639 Grenzach-Wyhlen, Germany

Drinking water ion exchanger and filter, pHresh, EMCO TECH Co. Ltd., Korea

Vitamin D and vitamin A were sporadically taken.

### 2.2. Procedure

The mentioned chemicals/dietary supplements have been taken as follows:

Alkalized drinking water was prepared *ad lib* by using water ion exchanger and filter. The filtered water was boiled prior to use.

Capecitabine was taken orally at 3.65 g Xeloda^®^/70 kg body weight per day. Two weeks of treatment were followed by one week of therapy pause per cycle.

“Budwig diet”: the following items were mixed for preparing a full batch using a blender: 1 kg Bio-Yoghurt, 0.1% fat, 0.25 kg Bio-Kefir, 1.5% fat, 6 table spoons of linseed oil, 4 table spoons of linseed, to be freshly milled: A part of this full batch may be prepared daily (the daily dose per person was about 250 grams).

Taken together around noon: 400 mg of Ubiquinol CoQ10 (4 capsules à 100 mg), 10 mg vitamin B2 (Riboflavin), 50 mg vitamin B3 (Niacin)

Taken three times daily: 2 softgels of MEGA EPA/DHA (eicosapentaenoic acid/docosahexaenoic acid), including 720 mg of EPA and 480 mg of DHA per 2 capsules.

One capsule of 5-Loxin^®^, one dose of Multinorm^®^ L-Carnitin aktiv (taken only during chemotherapy pause; during the chemotherapy 300 mg pure L-carnitine not containing vitamin B12 was ingested), one tablet of Unizink^®^ 50, and one tablet of Selenase^®^200 XXL were taken daily. EPA/DHA are COX-2 inhibitors. Therefore, the heart and vascular functions should be checked by a physician on a regular basis (it has been found that members of synthetic COX-2 inhibitors have been found to increase thrombosis, stroke, and heart attack risk under certain conditions). Moreover, Q10/B2/B3 were not taken in combination with radiation (the antioxidant Q10 potentially quenches the oxidative damage caused by radiation). EPA and DHA have potentially blood thinning effect.

## 3. Results

### 3.1. Applied Methodology and Methods

It has been hypothesized by the author that a multi-factorial approach towards breast cancer treatment would result in a synergetic response and reduced likelihood of development of resistance to treatment. Accordingly, it was sought to combine complementary, non-antagonistic treatments, which have the theoretical potential to suppress tumorigenesis and proliferation, with a “conventional” treatment. The envisaged therapy modules were Budwig diet and normalization of the fatty acid dietary balance, alkaline therapy, suppression of the inflammatory signaling chain, revitalization of the mitochondrial respiratory chain, bone protection against osteoclast-effected resorption by bisphosphonates and AKBA, and finally chemotherapy in the form of the prodrug capecitabine as 5-fluorouracil precursor [10]. The latter has been the recommended treatment by the medical tumor board in charge.

The described efforts have concretely been undertaken for suppressing refractory breast cancer stage IV in a female patient (body mass index 24–26, 41 years old), having developed a ductal carcinoma in *situ* in 2007. After biopsy revealed an estrogen receptor positive and progesterone receptor negative breast cancer, followed by surgical resection of the invaded sentinel lymph nodes, a neoadjuvant chemotherapy (four cycles Epirubicin/Cyclophosphamide, followed by four cycles of Taxotere^®^) was applied. However, the tumor showed little response (the tumor regression grade according to Sinn was only 1). Thus, the first and second axillary lymph node levels were resected in the following, and the affected breast was ablated. No suspicious tumor marker levels have been observed after ablation. The resection area was furthermore treated with radiation (gamma rays). The post-operational therapy included firstly tamoxifen, clodronate (a bisphosphonate), and a GNRH analogue (Enantone-Gyn^®^).

However, in September 2008, the patient - alerted by pain in the spinal cord - underwent MRI imaging, which revealed multiple bone metastases, including in the spinal cord.

As a consequence, the medication was altered as follows by the medical board in charge: Letrozol (aromatase inhibitor, 2.5 mg/d) and Ibandronat (6 mg intravenous infusion per month) as bisphosphonate. However, the disease progressed and a staging (^18^FDG-PET-CT and MRI) in March 2009 revealed the formation of various liver metastases. Therefore, the medication was changed to capecitabine chemotherapy instead of anti-hormonal therapy, accompanied by continuation of administration of Ibandronat.

Together with this therapy change, the author recommended the complimentary ingestion of the following substances: “Budwig diet” (linseed oil, flaxseed, and yoghurt), EPA/DHA concentrate in the form of distilled fish oil, ubiquinol (Q10 in reduced form), and vitamins B2 and B3, later on also 5-Loxin^®^ (AKBA). See above for further dosage and substance specifications.

### 3.2. Results

After about three months (June 2009) of continued intake of the above mentioned substances (besides 5-Loxin^®^), PET-CT showed no metabolic activity of the liver metastases any longer and reduced activity of the bone metastases under ^18^F-deoxyglucose as tracer in the PET. Concurrently, a decline of the tumor markers' (CA 15-3 and CEA) serum concentration was observed.

At this time, as a further element, 5-Loxin^®^ (AKBA) was introduced into the supplementation scheme for the reasons mentioned.

**Figure 1.**
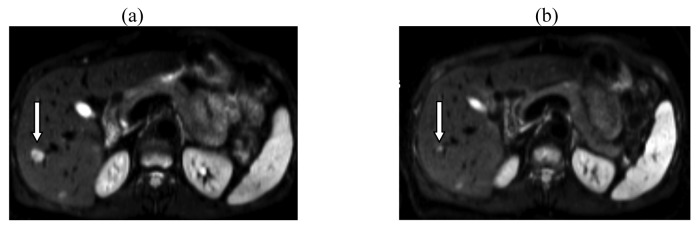
Diffusion-weighted MRI of the liver showing two metastases in the right lobe in (**a**) June 2009, and (**b**) February 2010. One metastasis (arrow) decreased from 15 mm in diameter to 7 mm, while the other remained unchanged (courtesy of Prof. Dr. E. Rummeny, Klinikum Rechts der Isar, Technische Universität München, Technical University of Munich, Germany).

Nine months later, the MRI showed that three out of six initial liver metastases could no longer be imaged, and that the largest lesion had decreased from about 15 mm to about 7 mm. A further small liver metastasis remained unchanged in size. This situation is depicted in Figure 1. Again, no metabolic activity in ^18^FDG-PET-CT was detected for any of the liver metastases.

Moreover, the PET-CT (^18^F-deoxyglucose as PET tracer) showed, in addition, a reduction of the size and metabolic activity of bone metastases, accompanied by re-calcification of the lesions. The response to treatment correlated with markedly decreased tumor marker serum levels, with CEA concentration being close to the significance threshold of 4 ng/mL. The development of the tumor marker levels over time is displayed in Table 1 below. The decline of tumor marker concentrations has been found to correlate with cancer remission in clinical studies on breast cancer patients [11,12]. In addition, the initial concentration of CEA has been associated with the clinical disease outcome in breast cancer patients.

The latest ^18^FDG-PET-CT of August 2010 showed ongoing sclerosis of at least some of the bone lesions and stable disease.

## 4. Discussion and Conclusions

A plethora of complementary cancer treatments have been reported. Firstly, the intake of polysaccharides and proteoglucans, such as mushroom and yeast glucans [ 13 , 14 ], mistletoe lectins [15,16] and nerium oleander extracts, the latter also in combination with sutherlandia frutescens extracts [17,18], have been described. The activation of the immune system against cancer cells has been ascribed to all of these compounds.

Another approach employed against the proliferation of cancer is alkaline therapy, which addresses the cellular acid-base balance. It has been found that extracellular/interstitial cancer tissue is more acidic than healthy tissue due to excessive production of lactic acid stemming from the glycolysis of glucose [19]. Otto Warburg already suggested in the last century that (as a consequence of hypoxia often encountered in tumor tissues) cancer cells undergo excessive glycolysis instead of relying on the energetically by far more effective oxidative phosphorylation [20,21], a fact which could recently also be verified by biopsy analysis in breast cancer patients, revealing a marked decrease in β-F1-ATPase/HSP60 expression ratio during disease progression [22]. Lately, it has been suggested that the initiation of glycolysis could be triggered by AKT activation during tumor development [23] and that the resulting acidification of the extra-cellular cancer tissue brings about survival advantages for cancer cells [9,24]. It has been found recently that T-cell development is markedly suppressed in acidified cancer tissue [38]. Alternative alkaline therapies applied for cancer treatment included the intake of sodium bicarbonate [25], cesium chloride [26], or alkaline diet, which is based on fruit and vegetables having high potassium content. A further approach was the ingestion of alkaline drinking water obtained from ion exchangers.

Another avenue towards cancer suppression has been established by the supplementation of (essential) polyunsaturated fatty acids, aiming at re-establishing cellular membrane functionality [27] and fluidity [28]. In addition, the polyunsaturated omega-3 fatty acids eicosapentaenoic (EPA) and docosapentaenoic acid (DHA) have been found to have a direct bearing on gene expression level by e.g., deactivation of NF-kappaB and AKT by EPA and DHA in a mouse model [29]. Polyunsaturated omega-3 fatty acids have also been shown to possess anti-inflammatory properties, for instance. by suppression of NF-KappaB and cyclooxygenases [30], or caused by the reduction of prostaglandin E2 biosynthesis via arachidonic acid due to a shift in the omega-6 fatty acid/omega-3 FA level towards omega-3 species (omega-6 fatty acids form the pool for the endogenous biosynthesis of E2 prostaglandin) [31,32].

In addition, a direct positive correlation between cytotoxic drug efficacy and DHA level in breast adipose tissue of patients has been observed [ 33 ]. Also, recent clinical studies suggested that EPA/DHA supplementation may suppress cancer-related cachexia [31]. Whereas severe side effects have been reported for the prolonged administration of some synthetic COX-II inhibitors, including increased thrombosis, stroke, and heart attack risk, to our best knowledge no comparably grave effects have been reported for the prolonged intake of EPA/DHA (e.g. in the form of fish oil) in clinical studies. The side effects of fish oil therapy, including blood thinning, have recently been discussed, e.g., by Farooqui *et al.* [34].

Likewise, Johanna Budwig established a cancer diet (the so-called “Budwig diet”), which includes *inter alia* the daily intake of linseed oil as a potent source of alpha-linolenic acid as essential omega-3 fatty acid [35]. Anecdotal cases of complete cancer remissions after continued Budwig diet have been reported [36]. To our best knowledge, no randomized clinical trials exploring the efficacy of the Budwig diet have been launched to date. The consequence of a continued Budwig diet is said to be an optimization of the dietary balance of omega-6/omega-3 fatty acids and reconstitution of physiologically intact cellular membrane composition by enhanced administration of polyunsaturated fatty acids as a substitute for peroxidized and saturated fatty acids in cellular membranes, thus increasing membrane fluidity. Furthermore, it has been hypothesized that polyunsaturated fatty acids may act as oxygen carriers [27]. The present-day Western diet results in an adverse ratio of about 15:1 of omega-6/omega-3 fatty acids, whereas a ratio of about 1:1 has been reported as paleolithic reference value for humans [34]. As a consequence, the endogenous high level of omega-6 fatty acids in humans fosters the increased biosynthesis of pro-inflammatory arachidonic acid from e.g., linoleic acid. Moreover, it has been hypothesized that cottage cheese, quark or yoghurt as second constituent of the Budwig diet refills the pool of sulfhydryl amino acids (which are essential for glutathione biosynthesis).

Warburg considered the glycolytic switch as being a final event in cancer formation, accompanied by irreversible genetic changes and the inactivation of the mitochondrial respiratory chain in cells, giving rise to their dedifferentiation [21]. However, recent studies suggest that this may not be the case: Dichloroacetate has been shown to be a potent inhibitor of pyruvate dehydrogenase kinase, thereby suppressing, as other agents, the glycolytic switch and thus fostering oxidative phosphorylation [37,38,39]. As a consequence of such an apparent normalization of the cellular energy production, cancer remissions in animal trials and anecdotal reports of healing of malignant tumors in human patients have been reported lately [40].

Moreover, investigations involving the administration of coenzyme Q10 directed towards the revitalization of the mitochondrial respiratory chain suggest that, indeed, the inhibition of the respiratory chain (Q10 is present in various complexes thereof) can be reversed or at least be halted: Folkers *et al.* reported that breast cancer patients taking 90 mg per day Q10 stayed in a state of constant disease, and did not develop new metastases. No patient in the group died, although about 20% (6/32) deaths were statistically expected in the observation period. When the dose of Q10 was augmented to 390 mg daily, five patients who already showed remission under 90 mg of Q10 per day went into apparently complete remission, including the eradication of liver metastases [41,42]. Cases of complete remission in response to high doses of Q10 for other cancer types, such as small cell bronchogenic carcinoma, have also been published by Folkers *et al.* [43].

Likewise, Sachdanandam *et al.* recently reported on tumor control and remission caused by a combination treatment by coenzyme Q10, vitamins B2 and B3 (all of which are essential for the cellular energy generation) and tamoxifen in animal trials [44]. As a result, markedly lower levels of lipid peroxidation and cachexia over the tumor-induced non-treated control group was observed. Orienting clinical trials of Premkumar *et al.*, involving 84 breast cancer patients, affirmed the anti-tumor action of said agent combination [45]. *Inter alia*, a decrease of the plasma concentration of urokinase plasminogen activator (UPA) by about 50% was observed, and the level of adhesion factors such as E-selectin and pro-angiogenic proteinase MMP-9 were found to be drastically decreased after only 90 days of treatment. Moreover, significantly reduced tumor marker levels (CA-15-3 and CEA) have been measured after 90 days of coenzyme Q10, vitamins B2 and B3 plus tamoxifen combination treatment [46]. UPA expression level was determined as correlating with the clinical outcome of breast cancer, and UPA inhibition has therefore been made the target of extensive research [47].

Another approach addressing the stabilization of the course of breast cancer is the administration of bisphosphonates [48] such as ibandronate, which stabilize the bone matrix and thus impede osteoclast-mediated bone lysis. In addition, certain bisphosphonates, such as the latter compound, have been shown to possess direct anti-tumor action *in vitro* and *in vivo* [49,50].

Finally, a further route towards the suppression of cancers is the suppression of nuclear factor kappa B (a gene transcription promoter involved in the inflammatory chain and in a tumor's capability to invade, metastasize and evade apoptosis) [51]. NF-kappaB stimulates the expression of various pro-inflammatory genes [52,53], also in breast cancer [54]. Consequently, a number of approaches have been divulged lately which are concerned with inhibition of this factor. The different compounds, which hinder the activation of NF-kappaB, are e.g., EPA (see above), and 11-keto-17-hydroxy boswellic acid (AKBA) [55], a compound which has been shown to abrogate the osteoclastogenesis by inhibition of NF-kappaB activation *in vitro*. AKBA was also shown to hinder the enzyme 5-lipoxygenase [56], which plays a pivotal role in the biosynthesis of pro-inflammatory leucotrienes. Remarkably, it has been shown that NF-kappaB inhibitors effectively inhibited MCF-7 breast cancer stem-like cells [57].

In the present case, refractory breast cancer, which had not or has poorly responded to initial chemo- and anti-hormonal therapy, showed drastic and ongoing response to a combination treatment including capecitabine and complementary treatment components; the latter include NF-kappaB blockers, and other inhibitors of the inflammatory chain, respiratory chain stimulants, plus alkaline therapy. The rationale for employing these agents has been explained in the preceding paragraphs. No resistance to the therapy was observed after 17 months, and the decrease of tumor marker levels correlated with imaging results. The obtained results are significant in view of the initial heavy disease progress and lack of relevant response to all preceding therapies.

The incremental contributions of each individual treatment element remain unclear. However, it is hypothesized that a synergetic action of the measures takes place. These have been selected by theoretical considerations in order to avoid potential antagonistic interferences, which could annihilate action. It should also be noted that concerns about the simultaneous intake of chemotherapeutics and antioxidants have been raised in the literature, especially in the context of cytostatics that have free radical formation as their believed primary mechanism of action. To our best knowledge, the primary mechanism of action of capecitabine, however, is not via free radicals but DNA synthesis and thymidylate synthase inhibition [10]. No antagonistic interaction with the remaining measures “base therapy” (addressing the immune suppression observed in the acidic tumor environment due to the purported suppression of T-cell development in the acidic tissue adjacent to tumors) and bone stabilization by bisphosphonates has been expected. On the contrary, the reported suppression of NF-kappaB expression by e.g. AKBA should reduce the RANKL-induced osteoclastogenesis, which is triggered by the transcription factor NF-kappaB [55].

Whether all measures contribute to the observed results remains speculative. The progression-free interval of 17 months observed so far is encouraging in view of a median time to progression from 3–9 months reported for the first line treatment of metastatic breast cancer by capecitabine [58]. Randomized clinical trials appear to be indicated in view of the promising orienting results.

Note that ER positive/PR negative breast cancer constitutes a rather limited high risk subset within the broader patient collective suffering from luminal breast cancer. Lately, it has been hypothesized in the literature that the expression of progesterone receptor (genes) in breast cancer has a positive bearing on the disease malignity and outcome, correlating with a less aggressive phenotype, and that the expression of progesterone receptor genes may be hindered by AP-1 [59,60,61]. AP-1 and NF-kappaB have been shown to bind to UPA promoter sequence and to cooperatively foster UPA expression. Consequently, it has been directly or indirectly suggested to therapeutically inhibit NF-kappaB in order to improve efficacy of antiestrogen treatment of patients associated to high risk hormone-dependent breast cancer [54,62].

Moreover, the reduced UPA expression mediated by Q10 described in the literature could also be a sign of a reduced activity of the transcription factor AP-1. At the same time, reduction of AP-1 activity could lead to a reversal of the blockage of the progesterone receptor expression caused by the inhibitory action of AP-1 and a consequent sensitization of ER-positive/PR-negative breast cancers to anti-estrogenic treatment by tamoxifen (compare to references [61,62]).

It is further hypothesized that the obtained orienting results hint (as already observed for DCA) at a revitalization of the mitochondrial respiratory chain at the expense of a pathologic increase of glycolysis. The reduction of glucose metabolism of the metastases was corroborated by reduced signal intensity in ^18^FDG-PET-CT scans during the treatment. Hence, the results are interpreted as a pointer towards the (at least partial) reversibility of the glycolytic switch and the associated changes in gene profile expression.

## Figures and Tables

**Table 1.** Development of the CEA and CA 15-3 serum concentrations over time; cut-off values were 4 ng/mL for CEA and 27 U/mL for CA15-3.

**Date/Months after Therapy Start**	**CA 15-3 (U/mL)**	**Excess over Cut-off Value [%]**	**CEA (ng/mL)**	**Excess over Cut-off Value [%]**
29 June 2009/3	49.3	82.6	31.4	684
13 September 2009/7	46.2	71.1	8.4	110
11 January 2010/10	37	37.0	4.1	2.5
19 April 2010/13	38.3	41.9	3.6	-10.8
12 July 2010/16	35.7	32.3	4.1	1.5

## Disclaimer

The author does not suggest that breast cancer can be healed by applying the described measures. Moreover, the author disclaims all responsibilities and liabilities as consequence of a potential application of the described treatment steps, either taken separately or in any combination by patients, third parties, institutions, or other persons, and for the correctness of the provided information. Questions concerning the disclosed treatment will be answered to physicians and clinical academia in general, only.
